# Detection of mobile genetic elements in multidrug-resistant *Klebsiella pneumoniae* isolated from different infection sites in Hamadan, west of Iran

**DOI:** 10.1186/s13104-021-05748-9

**Published:** 2021-08-26

**Authors:** Babak Asghari, Rezvan Goodarzi, Milad Mohammadi, Fatemeh Nouri, Mohammad Taheri

**Affiliations:** 1 Department of Medical Microbiology, Faculty of Medicine, Hamadan University of Medical Sciences, Hamadan, Iran; 2 Student Research Committee, Hamadan University of Medical Sciences, Hamadan, Iran; 3 Department of Pharmaceutical Biotechnology, School of Pharmacy, Hamadan University of Medical Sciences, Hamadan, Iran

**Keywords:** Mobile genetic elements, Multidrug-resistance, *K. pneumoniae*

## Abstract

**Objective:**

*Klebsiella pneumoniae* is one of most opportunistic pathogens that can be related to nosocomial infections. Increased acquisitions of multidrug resistance in this bacterium as well as the transfer of genes to other strains have caused concern. Integrons play key role in the acquisition and the spread of resistance genes. The aim of this study was evaluated the frequency of resistance genes *sulI*, *sulII*, *tetA*, *tetB,* class I (*intI* gene), class II integrons (*intII* gene) and the association between multidrug resistance and the presence of integrons in *K. pneumoniae*.

**Results:**

Antibiotics susceptibility test was performed on 126 of *K. pneumoniae* isolates. Also, DNA extraction was done and genes were detected using PCR method. In this study, 67 isolates (53%), carrying both the *sulI* and *sulII* genes. Forty-five percent tetracycline-resistant isolates were *tetA* or *tetB* positive. The prevalence of *intI* gene was 96%, while only sixteen isolate harboring *intII* gene (12.5%). Our results showed the high prevalence of integrons in MDR *K. pneumoniae*, indicating the important role of these genes in the transmission of antibiotic resistance.

**Supplementary Information:**

The online version contains supplementary material available at 10.1186/s13104-021-05748-9.

## Introduction

Klebsiella belongs to the Enterobacteriaceae family, which is known for causing human nosocomial infections such as urinary tract, intraabdominal, and upper respiratory tract infections (nosocomial pneumonia) [1, 2]. Genes encode capsule, lipopolysaccharide, siderophores, and pili, which are all known virulence agents in *K. pneumoniae*. Virulence factors have recently been discovered as iron absorption systems, efflux pumps, and a type VI secretion system [3].

Multidrug resistance has been increased globally that is considered public health threat. Several recent studies reported the emergence of multidrug-resistant bacterial pathogens from different origins including humans, poultry, cattle, and fish that increase the need for the proper application of antimicrobial agents as well as the routine application of the antimicrobial susceptibility testing to determine the antibiotic of choice [4–8]. Plasmids, transposons, and gene cassettes in integrons are examples of genetically engineered elements that contribute to antibiotic resistance [9].

Sulfonamides are antibiotics with a broad spectrum of action that are made chemically and used to treat bacterial infections [10]. In sulfonamide resistant strains, *sul* genes have been detected and provide translocation on chromosomes and plasmids are often associated with MGEs [11, 12]. Furthermore, plasmids harboring *sul* genes enable transfer among bacteria [13].

Integrons are genetic elements that include several genes and a special insertion site for the recombination system, enabling them to obtain mobile gene cassettes. Integrons are efficient systems in combining and expressing genes as part of their genetic elements known as gene cassettes [14, 15]. Based on the genes that encode integrase enzymes, five classes of integrons have been identified, with classes I, II, and III being the most frequent [16, 17].

This study was aimed to detect of mobile genetic elements in multidrug-resistant *K. pneumoniae *isolated from different infection sites in Hamadan, west of Iran.

## Main text

### Materials and methods

#### Bacterial isolates

One hundred twenty-six clinical isolates of *K. pneumoniae* were isolated from patients admitted to the hospitals from August 2019 to January 2021 in Hamadan, west of Iran. After colony morphology and gram staining, the biochemical tests including indole, citrate, urease, and Kligler iron agar (KIA) tests are employed and API method were applied [18].

#### Antimicrobial susceptibility testing

Antimicrobial susceptibility test was performed using disk diffusion method in Muller Hinton Agar medium, according to CLSI 2019 instructions. Antibiotics included Amoxicillin/clavulanic acid (30 μg), Cephalexin (30 μg), Ceftazidime (10 μg), Cefepime (30 μg), Ceftriaxone (30 μg), Cefoxitin (30 μg), Sulfamethoxazole/Trimethoprim (SXT 125/23.75 μg), Imipenem (10 μg), Aztreonam (30 μg), Tetracycline (30 μg), Moxifloxacin (5 μg), Levofloxacin (5 μg), Ciprofloxacin (10 μg), Gentamicin (10 μg), Amikacin (30 μg), Tigecycline (15 μg). *K. pneumoniae* ATCC 3565 was the corresponding control in the experiments Antibiotics Classification used in the present study was shown in Additional file 1.

#### DNA extraction and detection of genes

The isolates were cultured in LB broth and incubated at 35 °C overnight. The DNA concentration was determined using nanodrop. DNA amplification was performed in a thermal cycler (Apply biosystem, USA) using Mastermix (BioFact-Korea). The primers used in this study displayed in Table 1.

**Table 1:** Primer sequences used in this study

Gene	Primers sequence	Band size (bp)	References
*tet A*	F: GTGAAACCCAACATAC CCC R: GAAGGCAAGCAGGATG TAG	888	[27]
*tet B*	F: CCTTATCATGCCAGTCTTGC R: ACTGCCGTTTTTTCGCC	774	[27]
*SulI*	F: CGGCGTGGGCTACCTGAACG R: GCCGATCGCGTGAAGT TCCG	433	[36]
*SulII*	F: GCGCTCAAGGCAGATGGCATT R: GCGTTTGATACCGGCACCCGT	293	[36]
*IntI*	F: 5'-GCCTTGCTGTTCTTCTACGG-3' R: 5'-GATGCCTGCTTGTTCTACGG-3'	558	[31]
*IntII*	F: 5′-CACGGATATGCGACAAAAAGGT-3′ R: 5′-GTAGCAAACGAGTGACGAAATG-3′	789	[31]

#### PCR-based detection of antimicrobial resistance genes in the recovered *K. pneumoniae* isolates

The PCR reaction mixture contained 1 μL (10 pmol) of each primer, 2 μL DNA, 25 μL PCR Master Mix in a total 50 μL reaction volume. DNA amplification was conducted in a thermal cycler (Bio-Rad, USA), under the following conditions: initial denaturation at 94 °C for 5 min, followed by 30 cycles of denaturation at 94 °C for 30 s, annealing temperature for each gene for 40 s, an extension at 72 °C for 45 s, followed by a final extension at 72 °C for 4 min. The amplified DNA fragments, along with a 100 bp DNA size marker was run on 2% agarose gel electrophoresis.

#### Statistical analysis

The t-test was used to compare categorical results. All statistical tests were two-tailed, and statistical significance was defined as a P-value of 0.05. The statistical software package SPSS version 22 (IBM, NY) was used to analyze the data.

### Results

#### Antibiotic resistance pattern

**Fig. 1:**
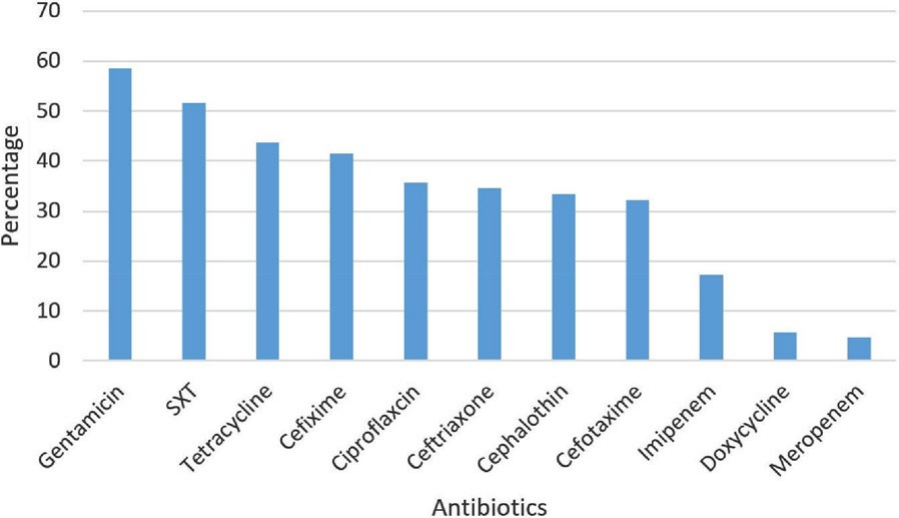
The highest frequency of resistance to antimicrobial agents

**Fig. 2:**
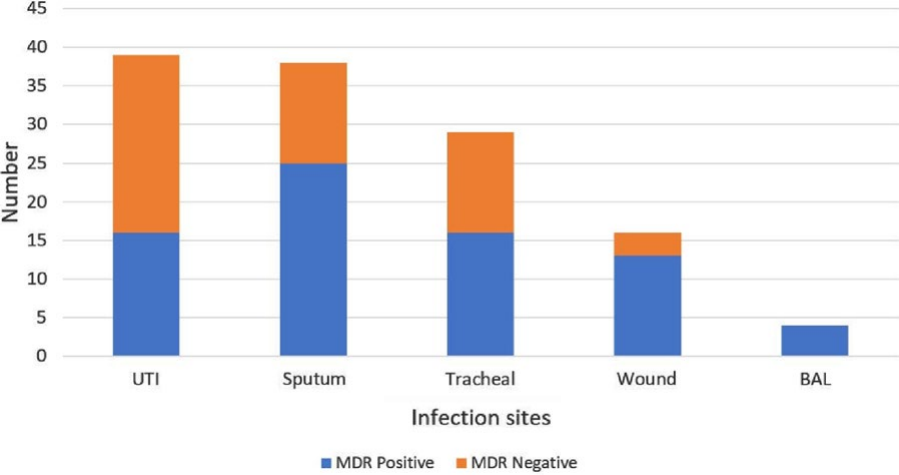
The frequency of different samples sites

Frequency of samples obtained were UTI 39 (31%), Sputum 38 (29.9), Tracheal 29 (23%), Wound 16 (12.6%) and BAL 4 (3.4%). The highest frequency of resistance to antimicrobial agents related to gentamicin (58.6%) and lowest to meropenem (4.6%) is shown in Fig. 1. In the present study, MDR was predominant among *K. pneumoniae* isolates because 58.6% of the isolates were MDR. The frequency of ESBL producing isolates was 52.9%. The frequency of samples in different parts of the body is shown in Fig. 2 according to MDR and non-MDR classification.

#### Frequency of genes and antibiotic susceptibility test

In this study, 51.7% of isolates were resistant to sulfamethoxazole/trimethoprim (SXT) which of 60.9% isolates harboring *sulI* and 26.4%had *sulII* gene. A significant relationship was seen only between *sulI* and SXT resistance (P = 0.008).

Fifty-five (43.7%) isolates were tetracycline resistant; among them 23% and 12.6%, isolates were positive for *tetA* and *tetB* genes, respectively. There was a significant relationship between the presence of these genes and tetracycline resistance (P < 0.01).

The prevalence of *int1* and *int2* genes among isolates were 102 (81.6%) and 50 (40.2%), respectively. The relationship between the presence of *int2* gene with ESBL producing, *sulI, tetA,* and MDR isolates was significant. The presence of *int2* gene and resistance to all antibiotics used in this study was significant (P < 0.01). The relationship between MDR and ESBL producing isolates with resistance to all antibiotics used in this study was significant (P < 0.001).

### Discussion

Due to the increase in antibiotic resistance, awareness of microbial resistance pattern and mechanisms of resistance transmission among bacterial infections can be an effective strategy to prevent microbial resistance transmission [19].

This study showed that there was a high level of antimicrobial resistance among *K. pneumoniae* isolates. More than 30% of the 126 *K. pneumoniae* isolates tested in this study demonstrated resistance to antimicrobials such as sulfamethoxazole/trimethoprim, tetracycline, ciprofloxacin, gentamicin, tetracycline, cefixime, cephalothin, ceftriaxone, and cefotaxime. The results of our study are consistent with the results of many past studies, which show a relatively high prevalence of *K. pneumoniae* resistant isolates in hospital settings. Moghadampour et al. showing resistance to gentamicin (30%), ceftazidime (34%), sulfamethoxazole/trimethoprim (22%), and ciprofloxacin (27%) have been reported in Iran [20].

In this investigation, 58.6% of *K. pneumoniae* isolates were MDR, which is higher than what has been reported in Kenya [21], but lower than what has been reported in China [22]. The results of this study, antibiotic resistance are similar to the results of Zomorodi et al. [23] in 2019, although the production of ESBL gene in our study is 52.9%, which shows an increase compared to their study which could indicate the transfer of genetic elements between bacteria and increased resistance to the ESBL gene.

In a 2021 study by Fatima et al.[24], among SXT-resistant strains, the frequency of *sulI* (66.7%) gene was similar to our study (60.9%). Although all of the tetracycline-resistant strains had the *tetB* gene, in our study only there was 12.6% of *tetB* gene that could indicate other mechanisms in the development of tetracycline resistance in bacteria.

The majority of MDR *K. pneumoniae* examined tested were positive for integron class 1. A high correlation has been established between the presence of integron class 1 and the prevalence of MDR in gram-negative bacteria. In a study of Li et al., integron 1 positive isolated bacteria showed a much higher incidence of drug resistance than negatively ones [25]. Other studies have shown a high prevalence of positive integron MDR-*K. pneumoniae*. The high prevalence of integron among MDR strains maybe since integron has the advantage of selective stress selection of strains in environments such as hospitals, which are caused by antibiotic abuse [26].

In our study, beta-lactam resistance was 88%, which compares with the results reported by Khamesipour et al. [27]. Tetracycline resistance is a common resistance among bacteria and its resistance was 43.7% among our isolates. In quinolone (ciprofloxacin) resistance (35.6%) among our isolates, they are much higher than reported in China [28], Iran [27], and India [29]. Aminoglycoside resistance has been reported to be the highest among antibiotics with a resistance rate of 58.6%. The data related to Tanzania and Kenya were higher for gentamicin compared with our study.

The result of PCR assay for tetracycline-resistant *K. pneumoniae* isolates and the frequency of genes reported to *tetA* or *tetB* was 23% and 12.6%, respectively. In Iran, Khamesipour et al. reported that *tetB* percentage (64.1%) and a higher percentage of *tetA* (79.4%), while the results of Bocaine et al. showed that all tetracycline-resistant *K. pneumoniae* isolates contained both the *tetA* and *tetB* genes. Previous studies have shown that *tetB* was in highly motile genetic elements that are easily transmitted between different bacterial genera. The association between tetracycline resistance genes (*tetA* and *tetB*) and class II integrons (*intII*) among our isolates suggests that class II integrons may be associated with the release of both tetracycline resistance genes. Similarly, Rezaei et al. reported a positive association between the presence of *intI* and *intII* genes and tetracycline resistance [30]. Our results show that integrons are widespread in isolated *K. pneumoniae*. Among the 126 isolates were int1 (81.6%) and int2 (40.2%), indicating high integrons presence. In this study, a significant relationship was also found (P < 0.001) between MDR phenotype and integrons, while Martinez Freijou et al. only described the tendency to develop resistance to several antimicrobial agents in strains with integrons. In this study, 81.6% of class I integron strains was observed, Derkhshan et al. that I integron was 25.8%, which shows a significant difference that can be found in the Derkhshan et al., of the majority of *K. pneumoniae* were isolated from urine [31]. In the present study, the integron class II with all antibiotics used was statistically significant, which could indicate antibiotic resistance due to integron class II. The prevalence of class 2 integrons in our MDR *K. pneumoniae* isolates was 40.2%, which is higher than that described by Rezaei et al. [30] and Firoozeh et al. [32] in northwestern and central Iran.

The frequency distributions of both *sulI* and *sulII* genes among sulfamethoxazole/trimethoprim-resistant isolates were 60.9% and 26.4%, respectively. This may be due to the widespread release of class 1 integron, which are closely related to the *sulI* gene.

As a common factor in the widespread dissemination of antimicrobial resistance genes, the prevalence of class 1 integrons has been reported to be 22 to 59%. In this study, the prevalence of class 1 integrons among clinical isolates of *K. pneumoniae* was 90%. Approximately similar frequencies of class 1 integrons have been reported in India (92%) and in China (93.2%). On the other hand, Class 1 integrons are less prevalent in other parts of world, including Brazil (65.5%) [33], Iran (66.6%) [27], Australia (73%) [34], United States (78.5%). and Korea (73.3%) [35].

Our study found a strong association between the presence of class II integrons and resistance to all antibiotics (P < 0.01). Class II integrons have generally been reported to be less common in some gram-negative organisms.

Monitoring the changes of integron gene cassettes in *K. pneumoniae* population can prevent the spread of antibiotic resistance factors in hospitals. Periodic monitoring and identification of these elements can help reduce disease burden, reduce costs, and shorten hospital stays. Overall, this study showed that carbapenems and doxycycline are the most effective antibiotics against *K. pneumoniae*. The high frequency of class I and II integron and the presence of MDR *K. pneumoniae* isolates is a serious warning for health authorities.

### Conclusion

Statistical relationships between drug resistance and integrons show that integrons are the encoder and disseminator of drug resistance among *K. pneumoniae* isolates. Mobile genetic elements are undeniable among bacteria as a natural phenomenon and bacteria become resistant to antibiotics in this way, it is necessary that with rational use of antibiotics also modifications in the pattern of antibiotics administered by physicians, be applied periodically.

## Limitation

Due to financial and time constraints in this research, the evaluation of several genes and also achieving more samples in various geographical locations in the country didn’t done and are among the limitations of this study. PCR-based detection of the most common virulence genes of *K. pneumoniae* should be carried out.

## Supplementary Information

**Additional file 1.** Antibiotics Classification used in the present study.

## Data Availability

The datasets used and/or analyzed during the current study available from the corresponding author on reasonable request.

## Abbreviations

CLSI: Clinical and laboratory standards institute
TSB: Tryptic soy broth
OD: Optical density
PCR: Polymerase chain reaction
