# Supplementary material for: Detection of mobile genetic elements in multidrug-resistant Klebsiella pneumoniae isolated from different infection sites in Hamadan, west of Iran
Source: BMC Res Notes. 2021 Aug 26;14:330. doi: 10.1186/s13104-021-05748-9 (PMC8394604; doi:10.1186/s13104-021-05748-9)
Supplement: Supplementary file 1 — Additional file 1. Antibiotics Classification used in the present study. [file 13104_2021_5748_MOESM1_ESM.docx]

Additional file 1. Antibiotics Classification used in the present study

| Antibiotic Classification | Mechanism | Antimicrobial Agents |
| --- | --- | --- |
| Sulfonamide | DHFRI | Trimethoprim/ sulfamethoxazole (SXT) |
| Cephalosporin | Inhibits cell wall synthesis | Ceftriaxone  Cefotaxime  Cefixime  Cephalothin |
| Penicillin | Inhibits cell wall synthesis | Imipenem  Meropenem |
| Tetracycline | Protein synthesis inhibition (30 s) | Doxycycline  Tetracycline |
| Fluoroquinolones | Nucleic acid synthesis inhibition | Ciprofloxacin |
| Aminoglycoside | Protein synthesis inhibition (30 s) | Gentamicin |

Abbreviation: DHFRI, dihydrofolate reductase inhibitors.
